# Effects on the yield and fiber quality components of *Bt* cotton inoculated with *Azotobacter chroococcum* under elevated CO_2_

**DOI:** 10.7717/peerj.15811

**Published:** 2023-08-07

**Authors:** Zhuo Li, Min Gao, Rujie Liu, Chunyan Chang, Feng Ge

**Affiliations:** 1 Institute of Plant Protection, Shandong Academy of Agricultural Sciences, Jinan, China; 2 State Key Laboratory of Integrated Management of Pest Insects and Rodents, Institute of Zoology, Chinese Academy of Sciences, Beijing, China; 3 College of Plant Protection, Shandong Agricultural University, Taian, China

**Keywords:** *Azotobacter chroococcum*, Bt cotton, Elevated CO_2_, Fiber quality, Yield

## Abstract

**Background:**

The raising trend of cultivation of *Bacillus thuringiensis* (*Bt*)-transgenic cotton is faced with a new challenge what effects on the growth and yield of *Bt* cotton under elevated CO_2_.

**Methods:**

Rhizobacteria is the significant biological regulator to increase environmental suitability and ameliorate soil-nitrogen utilization efficiency of crops, especially *Bt* cotton. Pot-culture experiments investigated the effects on the yield and fiber quality components of *Bt* cotton (transgenic Line SCRC 37) inoculated with *Azotobacter chroococcum* (AC) under elevated CO_2_.

**Results:**

The findings indicated that the inoculation of azotobacter significantly improved the yield and fiber quality components of *Bt* cotton, the elevated CO_2_ significantly increased the soil density of *A. chroococcum* and the partial yield indexes (as cottonweightper 20 bolls, lint yield per 20 bolls and boll number per plant), and non-significant decrease the fiber quality components of *Bt* cotton except uniform.

**Discussion:**

Overall results obviously depicted that the inoculation of azotobacter and the elevated CO_2_ had positive effects on the yield and fiber quality components of *Bt* cotton. Presumably, azotobacter inoculation can be used to stimulate plant soil-nitrogen uptake and promote plant growth for *Bt* cotton under elevated CO_2_ in the future.

## Introduction

With the increasing combustion of fossil fuels and the number of population, the concentration of atmospheric CO_2_ has increased by more than 40%, from 280 to 413 ppm, From the industrial revolution to the present (Douenne & Fabre, 2022). The recent forecast indicated that atmospheric CO_2_ concentration will increase to approximately 900 ppm by 2,100 (Lahn, 2021). It was the positive influence of eCO_2_ (elevated CO_2_) on crop production through affecting plant physiology and biochemistry, meanwhile, the eCO_2_ may cause a decline in nutritional quality (Cornelissen, 2011; Ainsworth & Rogers, 2007). Moreover, some research reported that the nitrogen metabolism could affect the nitrogen-related substances and enzyme activities (*e.g*., GPT and protease activity) for stimulating nutrient uptake to improve the growth and quality of *Bt* cotton (Devi et al., 2020; Devi, Singh & Thakral, 2021).

Nitrogen (N) plays a critical role in the physiology and biochemistry of plants (Richardson et al., 2009). Most of the nitrogen in nature is explored in the form of nitrogen gas (N_2_) which constitutes about 78% in the atmosphere and can’t be used directly by crops, crops usually use the available nitrogen directly from fertilizers in soil (Li, Parajulee & Chen, 2018). Azotobacter play a significant role in plants’ nitrogen cycle contributing to transform nitrogen gas into ammonia (Yamprai, Mala & Sinma, 2014). Therefore, rational application of nitrogen-fixing bacteria is a potential technique of biological regulator for optimizing soil-nitrogen management instead of the chemical fertilizers (Li, Parajulee & Chen, 2018).

*Azotobacter* sp. is the most ubiquitous free-living bacteria in plants’ rhizosphere which can fix nitrogen and enhance cotton growth (Khaitov et al., 2019; Chauhan et al., 2012). Some studies showed that *Azotobacter chroococcum* was the ubiquitous azotobacter in cotton, as well, it could improve cotton growth and nutrient uptake against chemical fertilization, especially 12 weeks after plant germination (Romero-Perdomo et al., 2017). Many researches had similar conclusions that the utilization of *A. chroococcum* inoculation could enhance the growth and grain yield of cotton (Khaitov et al., 2019; Shuppar & Kamal, 2021). Additionally, transgenic *Bacillus thuringiensis* cotton (*Bt* cotton), *Gossypium hirsutum*, have been the most abundant transgenic crop against target lepidopteran insects by expressing the Cry insect-resistant gene and been closely related to the total production of the cotton around the world (Lu et al., 2021). The azotobacter inoculation can accelerate roots nitrogen uptake to optimize the carbon nitrogen ratio with increasing the expression of nitrogen metabolism-related substances, enzyme activities and exogenous gene in *Bt* crops. Thus, we chose *Bt* cotton seeds inoculated by *Azotobacter chroococcum* to regulate the soil-nitrogen utilization efficiency and biomass nitrogen to improve the yield and quality of plants competing with the environmental stresses. In this research, we investigated the yield and fiber quality components of *Bt* cotton (transgenic line SCRC 37 with *Cry1Ab/Cry1Ac*) inoculated with *A. chroococcum* under elevated *vs* ambient CO_2_.

## Materials and Methods

### CO_2_ levels

This experiment was conducted in CO_2_-controlled growth cabinets (*i.e*., CCGC; GXM-508C-4-CO_2_; Ningbo Jiangnan Instrument CO., Ningbo, China) at the Experiment Station of Cotton Research Center, Shandong Academy of Agricultural Sciences (SAAS) located in Linqing County, Shandong Province of China (36.51°N, 115.42°E) on 24 April of 2018 and 2019. Two CO_2_ levels, ambient (400 μl/L, abbreviated as aCO_2_) and elevated (800 μl/L or double ambient, abbreviated as eCO_2_), were applied continuously during the experiment in both years. The CCGCs of elevated CO_2_ treatments were inflated with canned 99.95% CO_2_ gas and automatically controlled by the infrared CO_2_ analyzer (Range: 0–2,000 ppm; Ventostat 8102; Telaire Co., St. Marys, PA, USA) (Guo et al., 2012).

### Cotton cultivar

The transgenic Bt cotton (Line SCRC 37 with *Cry1Ab*/*Cry1Ac*, abbreviated as Bt) was obtained from the Cotton Research Center, Shandong Academy of Agricultural Sciences. The cotton cultivar line had middle-mature duration (approximately 129 d) and was suited to grow in Northern China (Zhang et al., 2009). These cottons were grown in plastic cases (38 cm in diameter and 35 cm in height) laden with 22 kg sterilized soil and 12 g NPK compound fertilizer (N: P: K = 4: 1: 2) on 24 April of 2018 and 2019. Soil available nutrients of N, P and K were quantified as 0.19%, 16.74 mg/kg and 317.62 mg/kg, respectively.

### Inoculation of cotton seeds with *Azotobacter chroococcum*

The glass tubes (diameter × height = 1.5 cm × 10 cm) with lyophilized powder of *Azotobacter chroococcum* (No. 10006, abbreviated as AC) were provided by the Agriculture Culture Collection of China (ACCC), Institute of Agriculture Resources and Regional Planning. *A. chroococcum* were cultivated by the specific culture medium in constant temperature shaker (28 °C, 200 rpm) until the absorbance of AC (1.001) reached to 1.000 at 600 nm. The culture medium for *Azotobacter chroococcum* with the ph of 7.0–7.2 contains 10.0 g Sucrose or Mannitol, 1.0 g CaCO_3_, 0.5 g K_2_HPO_4_·3 H_2_O, 0.2 g MgSO_4_·7H_2_O, 0.2 g NaCl, 15–20 g Agar, 1,000.0 ml distilled water. Before the inoculation of azotobacter, the supernatant of culture medium was discarded after centrifugation and then re-suspended the liquid medium until the azotobacter density was 10^8^ copies per milliliter. The cotton seeds were immersed by two different inoculations liquid mediums, the one is used with *A. chroococcum* (abbreviated as AC), the other is used without *A. chroococcum* (abbreviated as CK). Then those cotton seeds with azotobacter inoculation was dried for 4 h in the sterile draught cupboard at 28 °C.

### Pot-culture experiment setup

There were two CO_2_ levels (aCO_2_ *vs* eCO_2_) and two azotobacter inoculations (AC *vs* CK) in this experiment, with 20 replicates for a total of 80 pots. Each case with four cotton seeds at 1.5 cm depth were randomly distributed in the field; the soil was sterilized with a high temperature sterilizer (121 °C) and brought to ambient temperature prior to sowing of cotton seeds. These pots were maintained in CCGCs of aCO_2_ and eCO_2_ under controlled conditions (T = 26 ± 1 °C, RH = 65 ± 5%, L: D = 14 h: 10 h) without applications of pesticide and herbicide during the experiment. The rhizosphere soil samples were randomly sampled from each pot at three stages: pre-plant stage (1 day before sowing), seedling stage (14 days after sowing) and maturity stage (1 day before harvest). Then, those samples were used for analyzing the relative rhizobacteria densities by qRT-PCR with the specific primer (Table 1). One-day prior to sowing measure of *A. chroococcum* density was 5.31 ± 0.04 10^5^ in 2018 5.27 ± 0.07 10^5^ in 2019, respectively (Table 2).

**Table 1:** Sequence specific primer of *Azotobacter chroococcum* (AC) for qRT-PCR.

Primer	Sequence (5′-3′)	GenBank accession	Description
AC-2	Forward: GTGACCCGAAAGCTGACTCC	EU693338.1	*A. chroococcum* NifH gene
	Reverse: CCACCTTCAGCACGTCTTCC		

**Table 2:** Rhizobacteria densities in rhizosphere soil of *Bt* cotton inoculated with *A. chroococcum* (AC) and culture medium (CK) under ambient and elevated CO_2_ at 1 day before sowing, seedling stage and maturity stage in 2018 and 2019.

Sampled stage	Impact factors		2018 (copies/g)	2019 (copies/g)
1 day before sowing	/	/	5.31 ± 0.04 10^5^	5.27 ± 0.07 10^5^
Seedling stage	aCO_2_	AC	9.18 ± 0.05 10^11^ a	9.13 ± 0.05 10^11^ a
		CK	5.32 ± 0.07 10^5^ b	5.34 ± 0.05 10^5^ b
	eCO_2_	AC	9.22 ± 0.11 10^11^ a	9.17 ± 0.13 10^11^ a
		CK	5.29 ± 0.05 10^5^ b	5.40 ± 0.03 10^5^ b
Maturity stage	aCO_2_	AC	9.22 ± 0.10 10^11^ a, B	9.23 ± 0.06 10^11^ a, B
		CK	5.36 ± 0.06 10^5^ b	5.34 ± 0.10 10^5^ b
	eCO_2_	AC	1.18 ± 0.04 10^12^ a, A	1.16 ± 0.03 10^12^ a, A
		CK	5.29 ± 0.11 10^5^ b	5.28 ± 0.06 10^5^ b

**Note:**

CO_2_ levels: ambient CO_2_ (aCO_2_) *vs* elevated CO_2_ (eCO_2_). Azotobacter inoculation: *A. chroococcum* (AC) *vs* the culture medium (CK). /: none; data in table are average ± SE; different lowercase and uppercase letters indicate significantly different between AC and CK under same CO_2_ level and between aCO_2_ and eCO_2_ for same type of azotobacter inoculation at same sampling date in same year by the t test at *P* < 0.05, respectively.

### Nitrogen metabolism-related substances and enzyme activities of *Bt* cotton

Five fully-expanded leaf samples measured to 1.00 g were randomly selected from *Bt* cotton inoculated with azotobacter treatments (AC *vs* CK) at squaring stage. These frozen samples were thoroughly homogenized into the extraction buffer by fitted pestles, and then the supernatants were respectively collected into a 1.5 ml microcentrifuge tube after centrifugation. The content of free amino acids in samples were respectively quantified spectrophotometrically in contrast to a standard curve by ninhydrin colorimetry method (Jeong et al., 2023; Zhang, 1992). Then the soluble protein content and nitrogen metabolism-related enzyme activities of *Bt* cotton leaves were respectively determined by Coomassie Brillant Blue G-250 Dye Binding (Qiang et al., 2011; Ribeiro et al., 2013; Tosi & Ré, 2013) and Lal’s colorimetric method (Chen et al., 2014; Zhu et al., 2021).

### Yield and fiber quality components of *Bt* cotton

At maturity, ten plants were randomly selected from each treatment to record the yield characters *viz*., cotton weight per 20 bolls (g), lint yield per 20 bolls (g), boll number per plant and ginning outturn (%, abbreviated as GOT). Kapas were taken from the first picking to subject the fiber quality testing (*i.e*., length, strength, micronaire value, uniformity index and elongation) by using high volume instrument (HVI-900-A) at Institute of Cotton Research of Chinese Academy of Agricultural Sciences (CAAS), Anyang, Henan province of China (Nie et al., 2019).

### Data analysis

The data disposing and analyzing were used by SPSS Statistics 26.0 (IBM, Armonk, NY, USA, 2022).

Two-factor and Three-factor analysis of variances (ANOVAs) were used to test the influences of CO_2_ levels (aCO_2_ *vs* eCO_2_), azotobacter inoculation (AC *vs* CK), sampling years (2018 & 2019) and their interactions on the measured indexes of nitrogen metabolism-related substances (*i.e*., soluble protein and free amino acids content), enzyme activities (*i.e*., GPT and protease activity), yield (*i.e*., cotton weight per 20 bolls, lint yield per 20 bolls, boll number per plant and GOT) and fiber quality (length, strength, micronaire, uniform and elongation). The differences in average values of rhizobacteria densities, yield and fiber quality between cotton plants inoculated by AC and CK, between aCO_2_ and eCO_2_ were analyzed by t test at *P* < 0.05. The dissimilarity of those measured indexes between/among treatments was analyzed by the Duncan-test at *P* < 0.05.

## Results

### Incremental densities of *A. chroococcum* in cotton rhizosphere soil under elevated CO_2_ during different sampling stage

Inoculation with *A. chroococcum* (abbreviated as AC) have significant effects on the azotobacter density (*P* < 0.001), but non-significant influence (*P* ≥ 0.1, Table 3) of sampling year or any two- and three-interactions was measured on AC abundances at the seeding and maturity stages in each year (Tables 2 and 3). Compared to the ambient CO_2_, elevated CO_2_ raised obviously the AC density of the rhizosphere soil at cotton maturity stage; compared with culture medium without *A. chroococcum* (CK), we observed significant incremental trends of azotobacter density in the cotton root soil inoculated with AC during the seedling and maturity stages in 2018 and 2019 (*P* < 0.001; Tables 2 and 3). The interaction between CO_2_ and azotobacter inoculation was significant influence (*P* < 0.001) of the AC abundance in the soil samples at the maturity stage (Table 3).

**Table 3:** Three-way ANOVAs on the azotobacter density in rhizosphere soil of *Bt* cotton inoculated by *A. chroococcum* (AC) and culture medium (CK) under ambient and elevated CO_2_ at seedling and maturity stage in 2018 and 2019 (F/*P* values).

Impact factors	Seedling stage	Maturity stage
Y^a^	0.73/0.41	2.54/0.13
CO_2_^b^	0.48/0.50	4,837.79/<0.001***
Azoto^c^	119,688.61/<0.001***	348,288.08/<0.001***
Y × CO_2_	0.01/0.94	3.06/0.10
Y × Azoto	0.73/0.41	2.54/0.13
CO_2_ × Azoto	0.48/0.50	4,837.80/<0.001***
Y × CO_2_ × Azoto	0.01/0.94	3.06/0.10

**Notes:**

*** *P* < 0.001.

a Year (2018 *vs* 2019).

b CO_2_ level (aCO_2_ *vs* eCO_2_).

c Azotobacter inoculation (AC *vs* CK).

### Nitrogen metabolism-related substances and enzyme activities of *Bt* cotton inoculated with *A. chroococcum* under elevated CO_2_

Significant effects of azotobacter inoculation (*P* < 0.01 or *P* < 0.001) were observed on the soluble protein content, free amino acids content, GPT and protease activity in both years (Table 4). But non-significant effect (*P* > 0.05) of sampling year and CO_2_ treatment on these parameters except for GPT activity under CO_2_ treatment (*P* < 0.001, Table 4). Compared with culture medium without azotobacter, AC inoculation significantly increased the soluble protein content (+21.79%) and GPT activity (+63.27%) except for the free amino acids content (−1.86%) and protease activity (−2.22%) of *Bt* cotton, respectively (*P* < 0.01, Table 5).

**Table 4:** Three-way ANOVAs on the nitrogen metabolism-related substances and enzyme activities of *Bt* cotton inoculated by *A. chroococcum* (AC) and culture medium (CK) under ambient and elevated CO_2_ in 2018 and 2019 (F/P values).

Impact factors	Soluble protein content	Free amino acids content	GPT activity	Protease activity
Y^a^	2.24/0.15	2.22/0.16	0.06/0.81	0.002/0.97
CO_2_^b^	3.25/0.09	0.60/0.45	108.23/<0.001***	0.08/0.78
Azoto^c^	305.97/<0.001***	11.93/0.003**	3,531.30/<0.001***	61.40/<0.001***
Y × CO_2_	0.02/0.88	0.002/0.96	0.03/0.87	0.02/0.89
Y × Azoto	0.09/0.76	0.18/0.68	0.26/0.62	0.005/0.95
CO_2_ × Azoto	8.93/0.009**	1.32/0.27	283.72/<0.001***	12.80/0.003**
Y × CO_2_ × Azoto	0.04/0.85	0.05/0.84	1.26/0.28	0.04/0.84

**Notes:**

** *P* < 0.01.

*** *P* < 0.001.

a Year (2018 *vs* 2019).

b CO_2_ level (aCO_2_ *vs* eCO_2_).

c Azotobacter inoculation (AC *vs* CK).

**Table 5:** Nitrogen metabolism-related substances and enzyme activities of *Bt* cotton inoculated with *A. chroococcum* (AC) and culture medium (CK) under ambient and elevated CO_2_ in 2018 and 2019.

Impact factors	Factor levels	Soluble protein content (mg·g^−1^)	Free amino acids content (μmol·g^−1^)	GPT activity (μmol·g^−1^·h^−1^)	Protease activity (mg pro·g^−1^ FM)
CO_2_	Ambient	18.39 ± 1.66	340.25 ± 4.94	17.30 ± 3.26	1.82 ± 0.01
	Elevated	18.77 ± 2.27	341.65 ± 5.71	18.82 ± 5.83	1.82 ± 0.03
Azotobacter inoculation	AC	20.40 ± 0.71 a	337.82 ± 3.75 b	22.40 ± 2.11 a	1.80 ± 0.01 b
	CK	16.75 ± 0.47 b	344.09 ± 4.74 a	13.72 ± 0.56 b	1.84 ± 0.02 a

**Note:**

Data in table are average ± SE. Different lowercase letters indicate significantly different between azotobacter-inoculation treatments by the t test at *P* < 0.05.

**Figure 1:**
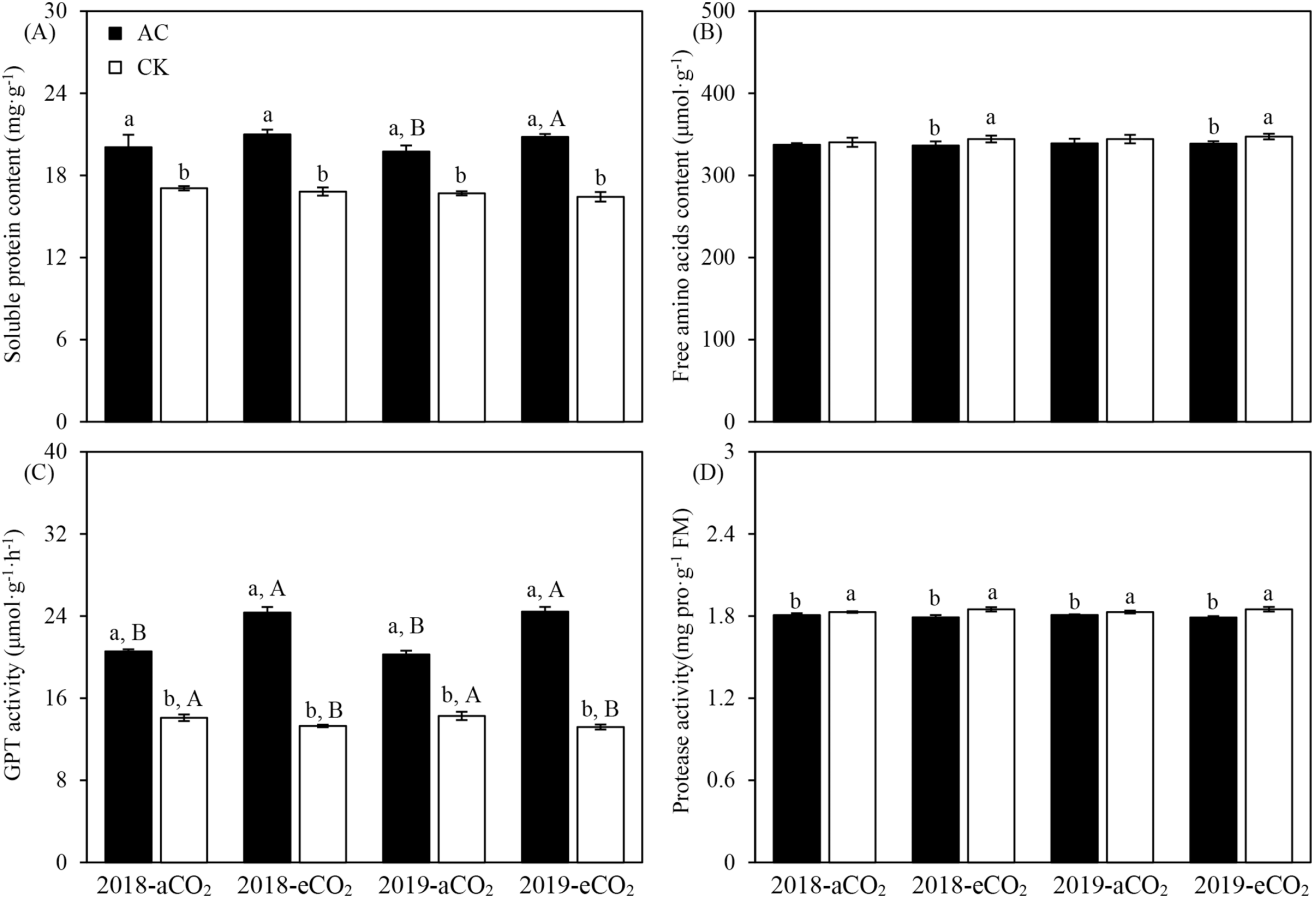
Three-factor interactions on nitrogen metabolism-related substances and enzyme activities of *Bt* cotton inoculated by *A. chroococcum* (AC) and culture medium (CK) under ambient CO_2_ (aCO_2_) and elevated CO_2_ (eCO). Each value indicates the average (+*SE*). Different lowercase and uppercase letters indicated significant difference between two azotobacter inoculations under same CO_2_ level in same year, and between aCO_2_ and eCO_2_ for same type of azotobacter inoculation in same year by the Duncan test at *P* < 0.05, respectively.

In addition to the significant main effects of azotobacter inoculation and CO_2_ treatment, there were significant interactions between two main impact factors on the soluble protein content, GPT and protease activity (*P* < 0.01 or *P* < 0.001) of *Bt* cotton except for the free amino acids content (*P* = 0.27 > 0.05) in both study years (Table 4). Compared with culture medium without azotobacter (CK), AC inoculation increased the soluble protein content (aCO_2_: +17.93%; eCO_2_: +25.69%) and GPT activity (aCO_2_: +43.86%; eCO_2_: +84.15%) except for the free amino acids content (eCO_2_: −2.48%) and protease activity (aCO_2_: −1.22%; eCO_2_: −3.31%) of *Bt* cotton (*P* < 0.05 or *P* < 0.001, Fig. 1); On the other hand, compared with ambient CO_2_, elevated CO_2_ significantly increased the soluble protein content (AC: +5.02%) of *Bt* cotton (*P* < 0.01, Fig. 1); Moreover, compared with ambient CO_2_, inverse trend was observed between two azotobacter inoculations in GPT activity (AC: +19.51%; CK: −7.11%) and protease activity (AC: −0.95%; CK: +1.10%) of *Bt* cotton growing under elevated CO_2_ (*P* < 0.05 or *P* < 0.001, Fig. 1).

### Yield of *Bt* cotton inoculated with *A. chroococcum* under elevated CO_2_

The yield characters of *Bt* cotton, including cotton weight per 20 bolls, lint yield per 20 bolls, boll number per plant and GOT, were significantly affected by azotobacter inoculation (*P* < 0.001, Table 6). Elevated CO_2_ treatment significantly influenced the measured indexes (*P* < 0.05 or *P* < 0.001) except for GOT (*P* = 0.88 > 0.05, Table 6). Compared with culture medium without azotobacter, AC inoculation significantly raised cotton weight per 20 bolls (+1.98%), lint yield per 20 bolls (+3.52%), boll number per plant (+26.55%) and GOT (+1.33%) of *Bt* cotton respectively (*P* < 0.001, Table 7). Moreover, elevated CO_2_ treatment increased cotton weight per 20 bolls (+1.35%) of *Bt* cotton compared with ambient CO_2_ (*P* = 0.02 < 0.05, Table 7).

**Table 6:** Three-way ANOVAs on the yield of *Bt* cotton inoculated by *A. chroococcum* (AC) and culture medium under ambient and elevated CO_2_ in 2018 and 2019 (F/*P* values).

Impact factors	Cotton weight per 20 bolls	Lint yield per 20 bolls	Boll number per plant	Ginning outturn
Y^a^	0.06/0.82	0.07/0.79	0.90/0.36	0.02/0.90
CO^b^_2_	185.71/<0.001***	62.50/<0.001***	8.10/0.012*	0.03/0.88
Azoto^c^	619.09/<0.001***	525.42/<0.001***	122.50/<0.001***	70.64/<0.001***
Y × CO_2_	0.20/0.66	0.07/0.79	0.10/0.76	0.22/0.64
Y × Azoto	0.29/0.60	0.30/0.59	0.10/0.76	0.04/0.84
CO_2_ × Azoto	63.62/<0.001***	37.99/<0.001***	0.90/0.36	3.37/0.04*
Y × CO_2_ × Azoto	0.29/0.60	0.18/0.68	0.10/0.76	0.01/0.91

**Notes:**

* *P* < 0.05.

*** *P* < 0.001.

a Year (2018 *vs*. 2019).

b CO_2_ level (aCO_2_ *vs*. eCO_2_).

c Azotobacter inoculation (AC *vs*. CK).

**Table 7:** The yield parameters of *Bt* cotton inoculated by *A. chroococcum* (AC) and culture medium (CK) under ambient and elevated CO_2_ in 2018 and 2019.

Impact factors	Factor levels	Cotton weight per 20 bolls (g)	Lint yield per 20 bolls (g)	Boll number per plant	Ginning outturn (%)
CO_2_	Ambient	118.72 ± 0.81 B	51.10 ± 0.69	12.08 ± 1.73	43.08 ± 0.29
	Elevated	120.33 ± 1.68 A	51.72 ± 1.19	12.83 ± 1.53	43.09 ± 0.38
Azotobacter inoculation	AC	120.70 ± 1.29 a	52.30 ± 0.60 a	13.92 ± 0.67 a	43.37 ± 0.17 a
	CK	118.35 ± 0.45 b	50.52 ± 0.14 b	11.00 ± 0.74 b	42.80 ± 0.14 b

**Note:**

Data in table are average ± SE. Different lowercase and uppercase letters indicate significantly different between azotobacter-inoculation treatments, and between CO_2_ levels by the t test at *P* < 0.05, respectively.

**Figure 2:**
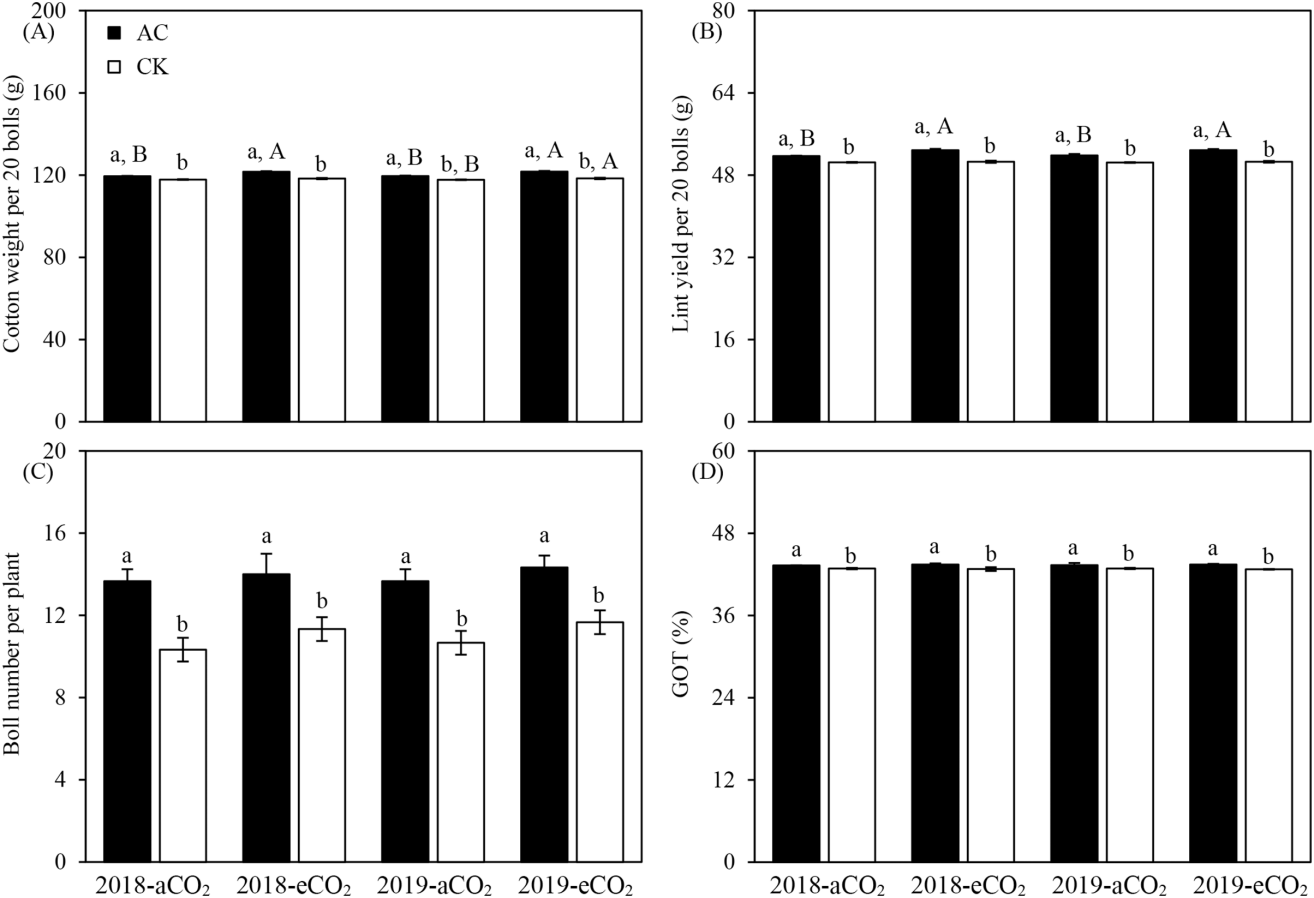
Three-factor interactions on the yield parameters of *Bt* cotton inoculated by *A. chroococcum* (AC) and culture medium (CK) under ambient CO_2_ (aCO_2_) and elevated CO_2_ (eCO_2_) in 2018 and 2019. Each value indicates the average (+*SE*). Different lowercase and uppercase letters indicated significant difference between two azotobacter inoculations under same CO_2_ level in same year, and between aCO_2_ and eCO_2_ for same type of azotobacter inoculation in same year by the Duncan test at *P* < 0.05, respectively.

In addition to the obvious influences of azotobacter inoculation and CO_2_ treatment, there were significant interactions between two main impact factors on cotton weight per 20 bolls, lint yield per 20 bolls and GOT of *Bt* cotton in both study years (*P* < 0.05 or *P* < 0.001, Table 6). Compared with culture medium without azotobacter (CK), AC inoculation increased the cotton weight per 20 bolls (aCO_2_: +1.46%; eCO_2_: +2.82%), lint yield per 20 bolls (aCO_2_: +2.58%; eCO_2_: +4.45%), boll number per plant (aCO_2_: +30.16%; eCO_2_: +23.19%) and GOT (aCO_2_: +1.10%; eCO_2_: +1.59%) of *Bt* cotton (*P* < 0.05 or *P* < 0.001, Fig. 2). On the other hand, compared with ambient CO_2_, elevated CO_2_ significantly increased the cotton weight per 20 bolls (AC: +1.84%; CK: +0.49%), lint yield per 20 bolls (AC: +2.11%), and boll number per plant (CK: +9.52%) of *Bt* cotton (*P* < 0.05, *P* ≤ 0.01 or *P* < 0.001, Fig. 2).

### Fiber quality components of *Bt* cotton inoculated with *A. chroococcum* under elevated CO_2_

There was significant influence of azotobacter inoculation (*P* < 0.001) on the fiber quality components of *Bt* cotton in both years (Table 8), but no significant influence (*P* > 0.1) of sampling year and CO_2_ treatment on the fiber quality parameters except for the uniform under CO_2_ treatment (*P* ≤ 0.04, Table 8). Compared with culture medium without azotobacter, AC inoculation significantly increased length (+7.64%), uniform (+1.70%), strength (+5.67%) and elongation (+2.26%) except for the micronaire (−5.54%) of *Bt* cotton, respectively (*P* < 0.001, Table 9).

**Table 8:** Three-way ANOVAs on the fiber quality of *Bt* cotton inoculated by *A. chroococcum* (AC) and culture medium under ambient and elevated CO_2_ in 2018 and 2019 (F/*P* values).

Impact factors	Length	Uniform	Strength	Micronaire	Elongation
Y^a^	0.02/0.89	0.79/0.39	0.12/0.74	0.64/0.43	0.67/0.43
CO^b^_2_	2.17/0.16	4.88/0.04*	0.04/0.85	0.07/0.79	0.09/0.76
Azoto^c^	159.85/<0.001***	159.47/<0.001***	166.09/<0.001***	52.07/<0.001***	73.31/<0.001***
Y × CO_2_	0.03/0.86	0.57/0.46	0.01/0.91	0.07/0.79	1.04/0.32
Y × Azoto	0.16/0.70	0.47/0.51	0.43/0.52	0.07/0.79	0.09/0.76
CO_2_ × Azoto	38.36/<0.001***	12.34/0.003**	15.46/0.001***	5.79/0.03*	15.00/0.001***
Y × CO_2_ × Azoto	0.002/0.99	0.33/0.57	0.001/0.99	0.07/0.79	0.26/0.62

**Notes:**

* *P* < 0.05.

** *P* < 0.01.

*** *P* < 0.001.

a Year (2018 *vs*. 2019).

b CO_2_ level (aCO_2_ *vs*. eCO_2_).

c Azotobacter inoculation (AC *vs*. CK).

**Table 9:** The fiber quality parameters of *Bt* cotton inoculated by *A. chroococcum* (AC) and culture medium (CK) under ambient and elevated CO_2_ in 2018 and 2019.

Impact factors	Factor levels	Length (mm)	Uniform (%)	Strength (cN•tex^−1^)	Micronaire	Elongation (%)
CO_2_	Ambient	28.26 ± 0.62	83.68 ± 0.61	28.30 ± 0.61	4.26 ± 0.10	6.73 ± 0.06
	Elevated	28.51 ± 1.68	83.93 ± 0.97	28.28 ± 1.09	4.27 ± 0.17	6.72 ± 0.11
Azotobacter inoculation	AC	29.43 ± 0.75 a	84.51 ± 0.43 a	29.07 ± 0.34 a	4.15 ± 0.08 b	6.80 ± 0.05 a
	CK	27.34 ± 0.53 b	83.10 ± 0.24 b	27.51 ± 0.37 b	4.38 ± 0.08 a	6.65 ± 0.05 b

**Notes:**

Data in table are average ± SE. Different lowercase letters indicate significantly different between azotobacter-inoculation treatments by the t test at *P* < 0.05, respectively.

**Figure 3:**
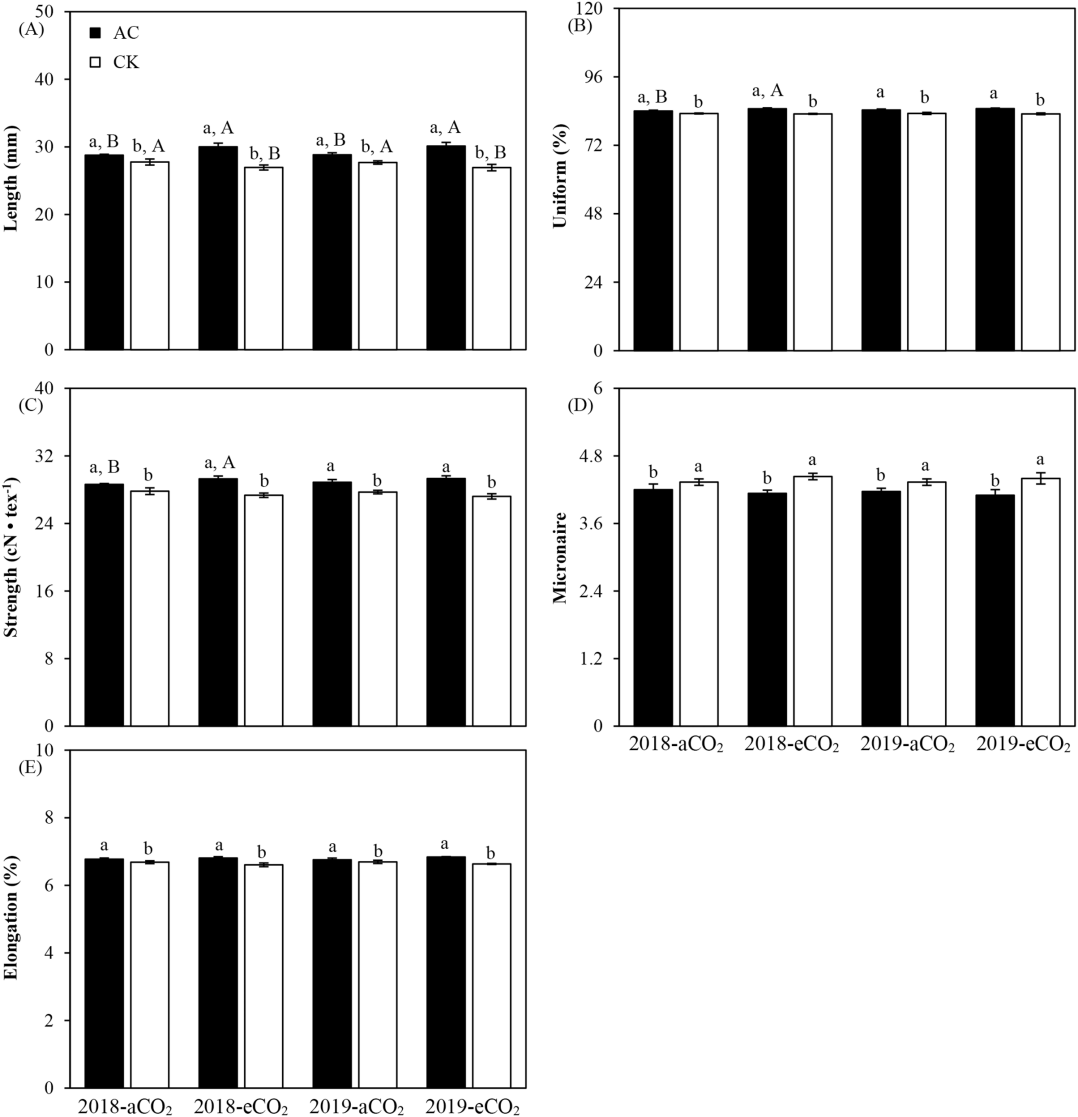
Three-factor interactions on the fiber quality parameters of *Bt* cotton inoculated by *A. chroococcum* (AC) and culture medium (CK) under ambient CO_2_ (aCO_2_) and elevated CO_2_ (eCO_2_) in 2018 and 2019. Each value indicates the average (+*SE*). Different lowercase and uppercase letters indicated significant difference between two azotobacter inoculations under same CO_2_ level in same year, and between aCO_2_ and eCO_2_ for same type of azotobacter inoculation in same year by the Duncan test at *P* < 0.05, respectively

In addition to the positive influences of azotobacter inoculation and CO_2_ treatment, there had significant interactions between two main impact factors on length, uniform, strength, micronaire and elongation of *Bt* cotton in both study years (*P* < 0.05, *P* < 0.01 or *P* ≤ 0.001, Table 8). Compared with culture medium without azotobacter (CK), AC inoculation increased length (aCO_2_: +3.86%; eCO_2_: +11.60%), uniform (aCO_2_: +1.23%; eCO_2_: +2.19%), strength (aCO_2_: +3.89%; eCO_2_: +7.44%) and elongation (aCO_2_: +1.15%; eCO_2_: +3.07%) of *Bt* cotton (*P* < 0.01 or *P* ≤ 0.001), except for micronaire (aCO_2_: −3.59%; eCO_2_: −7.29%) (*P* < 0.05, Fig. 3). On the other hand, compared with ambient CO_2_, elevated CO_2_ significantly increased the uniform (AC: +0.76%) and micronaire (CK: +1.92%), respectively (*P* = 0.03 or *P* < 0.05); Moreover, the inverse trend from ambient CO_2_ to elevated CO_2_ levels was observed between two azotobacter inoculations in length (AC: +4.42%; CK: −2.91%), strength (AC: +1.57%; CK: −1.82%) and elongation (AC: +0.86%; CK: −1.03%) of *Bt* cotton growing under elevated CO_2_ (*P* < 0.05 or *P* < 0.001, Fig. 3).

## Discussion

Transgenic *Bacillus thuringiensis* cotton (*Bt* cotton), *Gossypium spp*, hybrids expressing the Cry insect-resistant gene were adopted worldwide to control Lepidoptera pests (Luiz et al., 2021; Prasad, Rao & Naik, 2013), and were impressible to environmental variations, environmental stresses can cause changes on their growth and yield (Wang et al., 2015). The safe production of *Bt* cotton has attracted wide attention to their growth under climate change. Some studies indicated that the elevated CO_2_ have a positive influence on the yield and non-significant effect on the fiber quality of cotton (Wu, Chen & Ge, 2007; Reddy, Koti & Davidonis, 2004). In this study, we found the similar result that elevated CO_2_ treatment obviously raised the partial yield parameters of *Bt* cotton, including cotton weight per 20 bolls, lint yield per 20 bolls and boll number per plant, and fiber quality parameter as uniform with nitrogen metabolism compared to ambient CO_2_. Base on the “Nutrition compensation hypothesis”, the rising CO_2_ can directly change the biomass and photosynthetic rate of plants (Ainsworth & Rogers, 2007; Jackson et al., 2009; Zavala, Nabity & Delucia, 2013). Moreover, some studies indicated that elevated CO_2_ increased the carboxylation rates, which resulted in higher in the biomass of plant (Walker et al., 2020). In addition, nitrogen is recognized as a kind of non-carbon resource in nature, and nitrogen availability plays an important role in the effect of elevated CO_2_ on the biomass of plant (Du et al., 2020; LeBauer & Treseder, 2008). But limited studies focused on the yield and fiber quality components of transgenic *Bt* cotton inoculated by nitrogen-fixing bacteria competing with the environmental stresses.

**Scheme 1:**
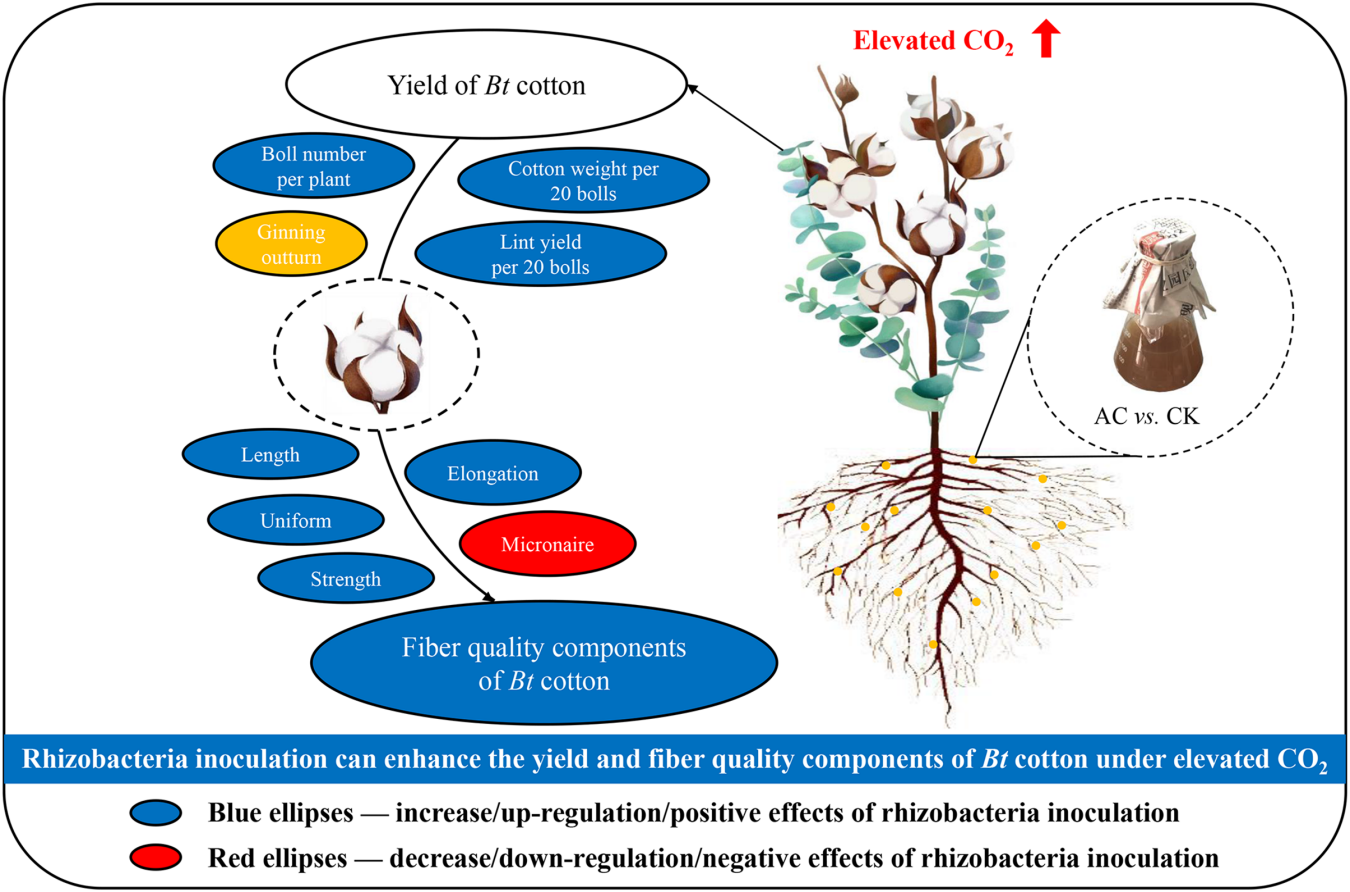
A schematic model that azotobacter inoculation can enhance the yield and fiber quality components of *Bt* cotton under elevated CO_2_.

Due to problems of environmental pollution and economic losses, lower nitrogen application rate and azotobacter inoculation have been largely researched to increase nitrogen use efficiency of crops (Bordoloi et al., 2019; Arora et al., 2018; Luo et al., 2018; Rodrigues, Ladeira & Arrobas, 2018). Therefore, we chose *Azotobacter chroococcum* inoculation of cotton seeds to regulate the soil-nitrogen utilization efficiency and create nutrient substances promoting the growth of crops to improve production as well as the quality of cotton at the same time. The results showed that azotobacter inoculation obviously have positive effects on the soluble protein content and GPT activity for stimulating the nitrogen metabolism to improve the yield and quality of *Bt* cotton against the environmental stresses. On the other hand, the elevated CO_2_ significantly increased the partial yield parameters (*e.g*., cotton weight per 20 bolls, lint yield per 20 bolls and boll number per plant) and uniform, suggesting that *A. chroococcum* inoculation might get compensatory nutrition for growth and production of *Bt* cotton competing with the environmental stresses under the sufficient soil nutrition; Moreover, inverse trends were observed between two azotobacter inoculations in the partial fiber quality parameters (*e.g*., length, strength and elongation) of *Bt* cotton growing under elevated CO_2_ consisting with variation tendency of GPT activity, attributing to azotobacter treatment improving the plants’ nitrogen uptake and utilization against the climate change. Overall consideration that significantly advantageous effects of elevated CO_2_ treatment on the yield and non-significant decline on the fiber quality parameter of *Bt* cotton, as well as azotobacter treatment promoting the growth, yield and quality of plants against the future climate change were observed when the *Bt* cotton inoculated with *A. chroococcum* under elevated CO_2_ (Scheme 1), which could be attributed to azotobacter stimulating soil-N uptake and compensatory nutrition of plants especially at elevated CO_2_ (Romero-Perdomo et al., 2017). Meanwhile, the *A. chroococcum* soil density at the maturity stage was increased with raising CO_2_, but there was non-significant difference of *A. chroococcum* soil density between elevated and ambient CO_2_ at the seedling stage. We speculate that the cotton root bifurcation and soil nutrient (*e.g*., carbohydrate, amino acid and microelements) for azotobacter with raising CO_2_ were enhanced to supply the scope and nutrition under long-term environmental influence. Chen et al. (2012) reported that there was a beneficial influence of elevated CO_2_ on the bacterial community in cornfield. Nitrogen availability including uptake and utilization was positively related to the growth of plant, and higher in nitrogen availability might be attributed to the rising CO_2_ (Newton et al., 2010). The rising CO_2_ could stimulated the fixtion rate of nitrogen which could increase nitrogen availability. In addition, elevated CO_2_ was reported to promote the accumulation of nitrogen only with nitrogen added extraly (Pastore, Megonigal & Langley, 2016). In our study, both the cotton weight per 20 bolls and lint yield per 20 bolls were increased by elevated CO_2_ only when the cotton seed inoculated with azotobacter. Therefore, we considered that elevated CO_2_ increased the biomass of cotton because of the higher nitrogen fixtion resulted from azotobacter. There was non-significance year-to-year variation (2018 *vs* 2019) in potted experiment data. These results explicitly indicated that *A. chroococcum* inoculation, as a potential technique of biological regulator, could raise the yield and fiber quality of *Bt* cotton by improving nitrogen metabolism competing with the environmental stresses (especially elevated CO_2_). In consequence, our research testified the novel approach of crop seeds inoculated with azotobacter as a biological nitrogen raising biomass N and maintaining a normal C/N ratio to improve the yield and quality of plants. The information propounded in our research will be particularly ponderable in developing strategies for transgenic crop sustainably and efficient production against atmospheric CO_2_ raising in the future.

## Conclusions

This study analyzed the yield and fiber quality components of *Bt* cotton inoculated with different azotobacter treatments under elevated and ambient CO_2_ in 2 years (2018 & 2019). Overall, our results expounded that there was significant positive influence on the partial yield and fiber quality components (*e.g*., cotton weight per 20 bolls, lint yield per 20 bolls, boll number per plant and uniform) and non-significant decline on the fiber quality parameter of *Bt* cotton under elevated CO_2_ in 2018 and 2019. Also, seed inoculation with *A. chroococcum* can stimulate the plants’ nitrogen uptake and utilization to promote the yield and quality of *Bt* cotton against the future climate change (especially elevated CO_2_) in both study years. Our research demonstrates that the use of *A. chroococcum*, as a potential technique of biological regulator, could overcome the environmental stresses to provide safe and sustainable production by enhancing soil-nitrogen utilization efficiency and biomass nitrogen of *Bt* crops.

## Supplemental Information

10.7717/peerj.15811/supp-1 Supplemental Information 1 The raw data.

## Additional Information and Declarations

### Data Availability

The following information was supplied regarding data availability: The raw data are available in the Supplemental File.
